# Trends in Emergency Department Visit Rates for Hypoglycemia and Hyperglycemic Crisis among Adults with Diabetes, United States, 2006-2011

**DOI:** 10.1371/journal.pone.0134917

**Published:** 2015-08-07

**Authors:** Jing Wang, Linda S. Geiss, Desmond E. Williams, Edward W. Gregg

**Affiliations:** Division of Diabetes Translation, Centers for Disease Control and Prevention, Atlanta, Georgia, United States of America; Hospital Universitario de Getafe, SPAIN

## Abstract

**Background:**

Despite concerns about hypoglycemia events from overly aggressive glycemic reduction, population trends in hypoglycemia and hyperglycemic crisis incidence are unclear. To address this gap, we examined changes in emergency department (ED) visit rates for hypoglycemia and hyperglycemic crisis 2006–2011.

**Methods:**

Using data from the Nationwide Emergency Department Sample, we estimated the number of ED visits for hypoglycemia and hyperglycemic crisis via ICD-9-CM among adults with diabetes. Using data from the National Health Interview Survey, we estimated the population of adults with diabetes and calculated ED visit rates.

**Results:**

From 2006 to 2011, ED visit rates for hypoglycemia declined by 22% from 1.8 to 1.4 per 100 adults (p = 0.003). The rates decreased in all age groups (all *P*<0.05) except those aged 18 to 44 years (*P* = 0.31). Hypoglycemia rates displayed a J-shaped curve across age, with the highest rates among adults aged 75 years or older (*P* <0.001). ED visit rates for hyperglycemic crisis did not change overall but increased 17% for adults aged 65 to 74 years (*P* = 0.02) and 29% for women (*P* = 0.01). Hyperglycemic crisis rates were highest among adults aged 18 to 44 years (*P* <0.001).

**Conclusions:**

Hypoglycemia rates have declined for all adults but persons aged 18–44 years while rates for hyperglycemic crisis remained stable. Future preventive efforts should target on the susceptible population of adults aged 18 to 44 years and those aged 75 years or older.

## Introduction

About 9.3% of the US population, or 29.1 million people, was estimated having diabetes in 2012 [1]. Preventing hypoglycemia from overly aggressive treatment for diabetic persons has been increasingly emphasized. Several clinical trials have shown that intensive glycemic reduction does not reduce cardiovascular events but increases hypoglycemia occurrence [2–4]. Hypoglycemia was found associated with higher cardiovascular morbidity and all-cause mortality [5,6]. These findings exposed the seriousness of hypoglycemia, and raised concerns on the harm of overly intensive glycemic intervention. Clinical guidelines for diabetes management were reconsidered to recommend a more flexible individualized approach that accounted for age, functional status, life expectancy, and morbidities into glycemic control goal setting [7,8]. Accordingly, American Diabetes Association and the Endocrine Society stated in a consensus report that preventing hypoglycemia can be considered more important than achieving optimal glycemic control for some subpopulations (e.g., older persons with comorbidities, children less than 5 years, or those with hypoglycemia unawareness) [9]. Despite these concerns over hypoglycemia, its national population trends are unclear. To address this gap, we examined the trends in emergency department (ED) visit for hypoglycemia and another major acute complication of diabetes-hyperglycemic crisis—from 2006 to 2011.

## Methods

We analyzed 2006–2011 data from Agency for Healthcare Research and Quality’s Nationwide Emergency Department Sample (NEDS) to identify ED visits for hypoglycemia and hyperglycemic crisis. NEDS is the largest all-payer ED database in the United States. It is a 20% stratified sample of EDs affiliated with community hospitals in the United States, containing about 25 to 30 million records annually from 24 to 30 partnering states. NEDS combines data from state emergency department databases for "treat and release" ED visits and from state inpatient databases for ED patients who are admitted to hospitals. NEDS contains information on patient and hospital characteristics, diagnosis and procedure codes, and the nature of visits. Nationally representative estimates can be derived by using sampling weights. Detailed information on the data is available at http://www.hcup-us.ahrq.gov/nedsoverview.jsp#Whatis. ED visits by adults aged 18 years or older with diabetes were identified via the *International Classification of Diseases*, *Ninth Revision*, *Clinical Modification* (ICD-9-CM) code 250.xx as any 1 of 15 diagnoses. Among ED visits by adults with diabetes, we identified ED visits for hypoglycemia on the basis of a previously validated algorithm [10,11] by using a first-listed diagnostic code of 251.0, 251.1, 251.2, or 962.3; or a first-listed diagnostic code of 250.8 that was not accompanied by any of the following codes: 259.8, 272.7, 681, 682, 686.9, 707.1, 707.8, 707.9, 709.3, 730.0, 730.1, 730.2, or 731.8. Visits for hyperglycemic crisis were defined as those with 250.1 or 250.2 as the first-listed diagnosis. The total unweighted number of visits was 401,804 for hypoglycemia and 197,415 for hyperglycemic crisis for 2006–2011 among adults with diabetes.

The number of adults with diagnosed diabetes in the general population was estimated from the 2006–2011 National Health Interview Survey (NHIS). The NHIS, conducted by the Centers for Disease Control and Prevention’s National Center for Health Statistics, is an annual household interview survey of a sample of the civilian, noninstitutionalized US population. Household response rates ranged from 80% to 87% for the 2006–2011 NHIS. Diabetes status was determined by response to the question, “Have you ever been told by a doctor or other health professional that you had diabetes?” The total unweighted number of adults with diagnosed diabetes was 14,838 for 2006–2011. Diabetes treatment status was determined by responses to questions about taking insulin or diabetic pills. The total unweighted number of adults with diagnosed diabetes who were being treated was 12,629 for 2006–2011.

We derived ED visit rates for hypoglycemia and hyperglycemic crisis by using the number of ED visits for each condition as the numerator and the number of adults with diagnosed diabetes as the denominator. We also conducted 2 separate sensitivity analyses on the trends in ED visit rates for hypoglycemia and hyperglycemic crisis by using all 15 diagnoses from NEDS and by restricting denominators to adults being treated for diabetes with either insulin or oral medications. ED visit rates were calculated by age group (aged 18−44 y, 45−64 y, 65−74 y, or ≥75 y) and by sex. ED visit rates were age-adjusted by using as the standard the 2010 NHIS population with diabetes in these 4 age groups.

Our study used secondary public data sources and was therefore exempted from institutional review board.

### Statistical Analysis

All analyses were conducted using SAS version 9.1.3 (SAS institute, Inc., Cary, North Carolina) and SUDAAN version 11.0.1 (Research Triangle Institute, Research Triangle Park, North Carolina) to account for the complex sample designs of NEDS and NHIS. We combined NEDS and NHIS data to derive ED visit rates and the associated standard errors and confidence intervals (CIs) based on the Taylor series linearization method. We conducted linear regression to test trends in ED visit rates or numbers from 2006 to 2011 with weighted least square method by the inverse of the variance of the estimates in each year. We used t-tests to compare the difference in percentage or ED visit rates between two groups. Significance was set at *P* <0.05 for 2-sided tests.

## Results

### Patient Characteristics

Among adults with diabetes, the weighted number of ED visits for hypoglycemia decreased from 308,232 in 2006 to 282,254 visits in 2011 (*P* = 0.0499) (Table 1), and the weighted number of ED visits for hyperglycemic crisis increased from 129,752 in 2006 to 174,998 in 2011 (*P* < 0.001). Among all ED visits by adults with diabetes, 3.3% were for hypoglycemia in 2006, which declined to 2.2% in 2011 (*P* < 0.001); hyperglycemic crisis accounted for 1.4% in both 2006 and 2011 (*P* = 0.97). About half of the hypoglycemia visits were by adults aged 65 years or older, while 58% of hyperglycemic crisis visits were by adults aged 18 to 44 years and only about 10% were by adults 65 years or older. Compared to hypoglycemia, a larger proportion of visits for hyperglycemic crisis led to hospital admission (87.6% vs 27.3% in 2011, *P* < 0.001), and a greater proportion of these patients died in the ED or hospital (0.5% vs 0.2% in 2011, *P* < 0.001).

**Table 1:** Characteristics of Emergency Department Visits for Hypoglycemia or Hyperglycemic Crisis Among Adults With Diagnosed Diabetes.

Characteristic	Hypoglycemia, n ^a^ (%)		Hyperglycemic Crisis, n ^a^ (%)	
	2006 (N = 65,407)	2011 (N = 63,972)	2006 (N = 27,627)	2011 (N = 39,718)
% of total ED visits by diabetic adults	3.3	2.2	1.4	1.4
Age, y				
18–44	11,440 (17.6)	10,439 (16.4)	17,146 (61.9)	22,947 (57.9)
45–64	20,780 (32.0)	21,672 (34.0)	8,111 (29.5)	12,869 (32.3)
65–74	13,264 (20.2)	13,472 (20.9)	1,281 (4.7)	2,290 (5.8)
≥75	19,923 (30.2)	18,389 (28.6)	1,089 (3.9)	1,612 (4.1)
Female sex	34,717 (53.1)	33,115 (51.6)	13,551 (48.9)	19,344 (48.6)
Expected primary payer (18–64 years)				
Medicare	8,712 (27.0)	9,143 (28.5)	3,486 (13.8)	5,890 (16.5)
Medicaid	6,858 (21.8)	7,734 (24.4)	6,215 (25.1)	10,085 (28.5)
Private insurance	10,704 (33.2)	9,024 (28.4)	7,786 (30.7)	9,591 (27.1)
Uninsured or other	5,842 (18.1)	6,113 (18.6)	7,696 (30.5)	10,135 (27.9)
Discharge disposition				
Treated and released	4,3965 (67.5)	43,261 (67.5)	1,692 (6.1)	4,362 (10.2)
Admitted	16,997 (25.7)	17,334 (27.3)	25,272 (91.4)	34,420 (87.6)
Other^b^	4,445 (6.7)	3,377 (5.2)	663 (2.5)	936 (2.2)
Died (in the ED or in the hospital)	150 (0.2)	139 (0.2)	186 (0.7)	190 (0.5)

Abbreviation: ED, emergency department. Data from the Nationwide Emergency Department Sample.

^a^Unweighted numbers.

^b^ Including transfers to skilled nursing facility, intermediate care, home health care, against medical advice, died in ED, and unknown destinations.

### ED Visit Rates for Hypoglycemia

Age-adjusted ED visit rates for hypoglycemia declined 22%, from 1.8 (95% CI, 1.7 to 1.9) per 100 diabetic adults in 2006 to 1.4 (95% CI, 1.3 to 1.5) per 100 adults with diabetes in 2011 (*P* for trend = 0.003) (Table 2). Similar declines were seen for both sexes (*P* = 0.01 for men and *P* = 0.002 for women) (Table 2). Age-specific ED visit rates for hypoglycemia decreased 22% (*P* = 0.03) for adults aged 45–64 years, 33% (*P* < 0.001) for those aged 65–74 years, and 22% (*P* = 0.02) for those aged 75 years or older, but there was no consistent trend for adults 18–44 years (*P* = 0.31) (Table 2).

**Table 2:** Emergency Department Visit Rates for Hypoglycemia and Hyperglycemic Crisis Among Adults With Diagnosed Diabetes, 2006–2011.

Characteristic	Rate per 100 (95% CI)						Percentage Change (95% CI)	*P* for Trend
	2006	2007	2008	2009	2010	2011		
**Hypoglycemia**								
Crude rate by age, y								
Overall	1.8 (1.7,1.9)	1.8 (1.7,2.0)	1.6 (1.5,1.8)	1.5 (1.3,1.6)	1.5 (1.4,1.6)	1.4 (1.3,1.5)	-23.9 (-31.8,-15.9)	**0.003**
18 to 44	1.9 (1.6,2.1)	2.2 (1.8,2.5)	2.0 (1.6,2.3)	1.4 (1.2,1.7)	1.7 (1.4,1.9)	1.7 (1.5,1.9)	-7.6 (-26.3,11.1)	0.31
45 to 64	1.3 (1.1,1.4)	1.3 (1.1,1.4)	1.1 (1.0,1.2)	1.0 (0.9,1.1)	1.1 (1.0,1.2)	1.0 (0.9,1.1)	-22.0 (-33.5,-10.6)	**0.03**
65 to 74	1.8 (1.6,2.0)	1.7 (1.5,1.9)	1.6 (1.4,1.8)	1.5 (1.3,1.7)	1.4 (1.2,1.5)	1.2 (1.1,1.3)	-32.8 (-43.1,-22.4)	**<0.001**
≥75	3.1 (2.7,3.6)	3.3 (2.9,3.8)	3.2 (2.8,3.7)	2.8 (2.4,3.1)	2.4 (2.1,2.6)	2.4 (2.2,2.7)	-22.3 (-36.1,-8.5)	**0.02**
Age-adjusted, by sex^a^								
Overall	1.8 (1.7,1.9)	1.9 (1.7,2.0)	1.7 (1.6,1.8)	1.5 (1.4,1.6)	1.5 (1.4,1.5)	1.4 (1.3,1.5)	-22.2 (-30.3,-14.2)	**0.003**
Men	1.8 (1.6,2.0)	1.8 (1.6,2.0)	1.7 (1.5,1.9)	1.4 (1.3,1.5)	1.4 (1.3,1.5)	1.4 (1.3,1.5)	-23.5 (-33.5,-13.5)	**0.01**
Women	1.8 (1.6,2.0)	1.9 (1.7,2.0)	1.7 (1.5,1.8)	1.6 (1.4,1.7)	1.5 (1.4,1.7)	1.4 (1.3,1.6)	-21.0 (-30.4,-11.6)	**0.002**
**Hyperglycemic crisis**								
Crude rate by age, y								
Overall	0.8 (0.7,0.8)	0.8 (0.7,0.8)	0.8 (0.7,0.8)	0.7 (0.7,0.8)	0.8 (0.7,0.8)	0.8 (0.8,0.9)	11.8 (0.2,23.4)	0.28
18 to 44	2.7 (2.3,3.2)	3.5 (2.8,4.1)	3.3 (2.7,3.9)	2.7 (2.3,3.1)	3.1 (2.7,3.6)	3.7 (3.2,4.2)	36.0 (8.9,63.1)	0.30
45 to 64	0.5 (0.4,0.5)	0.5 (0.4,0.6)	0.5 (0.4,0.5)	0.5 (0.4,0.5)	0.5 (0.5,0.6)	0.6 (0.5,0.6)	18.4 (-0.2,37.0)	0.11
65 to 74	0.2 (0.2,0.2)	0.2 (0.1,0.2)	0.2 (0.2,0.2)	0.2 (0.2,0.2)	0.2 (0.2,0.2)	0.2 (0.2,0.2)	16.7 (-0.1,33.4)	**0.02**
≥75	0.2 (0.1,0.2)	0.2 (0.1,0.2)	0.2 (0.2,0.2)	0.2 (0.2,0.2)	0.2 (0.2,0.2)	0.2 (0.2,0.2)	23.5 (5.2,41.9)	0.10
Age-adjusted, by sex^a^								
Overall	0.7 (0.6,0.8)	0.8 (0.7,0.9)	0.8 (0.7,0.9)	0.7 (0.6,0.8)	0.8 (0.7,0.8)	0.9 (0.8,1.0)	29.0 (10.4,47.5)	0.17
Men	0.7 (0.6,0.8)	0.9 (0.7,1.0)	0.8 (0.7,0.9)	0.7 (0.6,0.8)	0.8 (0.7,0.9)	0.9 (0.8,1.1)	28.4 (5.1,51.7)	0.44
Women	0.7 (0.6,0.7)	0.7 (0.6,0.8)	0.7 (0.6,0.8)	0.7 (0.6,0.8)	0.8 (0.7,0.9)	0.8 (0.7,0.9)	29.2 (7.5,50.9)	**0.01**

Abbreviation: CI, confidence interval.

Data from the Nationwide Emergency Department Sample and National Health Interview Survey (NHIS).

^a^ Age-adjusted to population with diabetes in 2010 based on NHIS using age groups 18–44 years, 45–64 years, 65–74 years, and ≥75 years.

Boldface indicates statistical significance (*p*<0.05)

ED visit rates for hypoglycemia displayed a J-shaped curve across age, with the highest rate in adults aged 75 years or older (2.4 (95% CI, 2.2 to 2.7) per 100 people in 2011), about twice the rates for adults 65–74 years (1.2 (95% CI, 1.1 to 1.3) per 100) and 45–64 years (1.0 (95% CI, 0.9 to 1.1) per 100); adults aged 18 to 44 years had the next highest rates (1.7 (95% CI, 1.5 to 1.9) per 100) (all *P* < 0.001 for pairwise comparisons between age groups). The rates were similar between men and women (*P* = 0.31) (Table 2).

### ED Visit Rates for Hyperglycemic Crisis

There was no discernable linear trend in overall age-adjusted ED visit rates for hyperglycemic crisis, with rates of 0.7 (95% CI, 0.6 to 0.8) per 100 persons in 2006 and 0.9 (95% CI, 0.8 to 1.0) per 100 persons in 2011 (*P* = 0.17) (Table 2). Although there was also no significant linear trend for men (*P* = 0.44) during the time period, ED visit rates for hyperglycemic crisis increased for women (*P* = 0.01) (Table 2). Age-specific rates increased 17% for adults 65–74 years (*P* = 0.02) but did not change significantly for other age groups (all *P* >0.05) (Table 2).

Rates of hyperglycemic crisis were highest among adults aged 18 to 44 years (3.7 (95% CI, 3.2 to 4.2) per 100 people in 2011) and much lower in older age groups (0.6 for those aged 45–54 y (95% CI, 0.5 to 0.6), 0.2 (95% CI, 0.2 to 0.2) for those aged 65–74 y, and 0.2 (95% CI, 0.2 to 0.2) for those ≥ 75 y) (*P* = 0.72 for the difference between 65–74 y and ≥75 y and *P* < .001 for all other comparisons between age groups). Thus the rates for adults aged 18–44 years were 6 to 18 times that of other age groups. The rates were similar between men and women (*P* = 0.12) (Table 2).

### Sensitivity Analyses

We conducted sensitivity analyses to examine whether trends in ED visit rates for both conditions differed when the denominator for rates was restricted to the respondents with diabetes who were being treated with insulin or oral medication. Although rates for the treated population were higher than for the overall population with diabetes, trends were consistent (Table 3). Further, when we used all listed diagnoses instead of only the primary diagnosis to define the conditions, trends overall and for most subgroups remained unchanged (data not shown).

**Table 3:** Emergency Department Visit Rates for Hypoglycemia and Hyperglycemic Crisis Among Adults With Treated Diabetes 2006–2011.

Characteristic	Rate per 100 (95% CI)						Percentage change	*P* for Trend
	2006	2007	2008	2009	2010	2011		
**Hypoglycemia**								
Crude rate, by age, y								
Overall	2.2 (2.0,2.4)	2.2 (2.0,2.3)	2.0 (1.8,2.1)	1.8 (1.6,1.9)	1.7 (1.6,1.8)	1.6 (1.5,1.7)	-26.1 (-34.2,-18.1)	**<0.001**
18 to 44	2.7 (2.2,3.2)	3.2 (2.6,3.7)	3.0 (2.3,3.6)	2.1 (1.7,2.4)	2.2 (1.9,2.6)	2.2 (1.8,2.5)	-19.3 (-38.1,-0.6)	0.10
45 to 64	1.5 (1.4,1.7)	1.5 (1.3,1.6)	1.3 (1.1,1.4)	1.2 (1.1,1.3)	1.2 (1.1,1.4)	1.2 (1.1,1.2)	-23.8 (-34.1,-13.6)	**0.02**
65 to 74	2.0 (1.8,2.3)	1.8 (1.6,2.1)	1.8 (1.6,2.0)	1.7 (1.5,1.9)	1.6 (1.4,1.7)	1.4 (1.3,1.5)	-30.9 (-41.8,-19.9)	**<0.001**
≥75	3.6 (3.1,4.1)	3.8 (3.2,4.4)	3.7 (3.1,4.2)	3.2 (2.8,3.6)	2.7 (2.4,3.1)	2.8 (2.5,3.2)	-22.1 (-36.4,-7.8)	**0.01**
Age-adjusted By sex^a^								
Overall	2.2 (2.0,2.3)	2.2 (2.0,2.4)	2.1 (1.9,2.2)	1.8 (1.6,1.9)	1.7 (1.6,1.9)	1.7 (1.5,1.8)	-24.0 (-32.2,-15.7)	**0.002**
Men	2.2 (1.9,2.4)	2.2 (1.9,2.4)	2.0 (1.8,2.3)	1.7 (1.5,1.8)	1.6 (1.4,1.8)	1.6 (1.4,1.7)	-27.6 (-37.7,-17.6)	**0.006**
Women	2.2 (1.9,2.4)	2.2 (2.0,2.5)	2.1 (1.8,2.3)	1.9 (1.7,2.1)	1.8 (1.7,2.0)	1.7 (1.6,1.9)	-19.9 (-30.7,-9.1)	**0.001**
**Hyperglycemic crisis**								
Crude rate, by age, y								
Overall	0.9 (0.8,1.0)	0.9 (0.8,1.0)	0.9 (0.8,1.0)	0.9 (0.8,1.0)	0.9 (0.8,1.0)	1.0 (0.9,1.1)	8.7 (-3.9,21.3)	0.38
18 to 44	4.0 (3.3,4.7)	5.0 (4.1,6.0)	5.0 (4.0,6.0)	3.9 (3.3,4.6)	4.3 (3.5,5.0)	4.7 (4,5.4.0)	18.8 (-8.2,45.9)	0.72
45 to 64	0.6 (0.5,0.6)	0.6 (0.5,0.6)	0.5 (0.5,0.6)	0.6 (0.5,0.6)	0.6 (0.6,0.7)	0.7 (0.6,0.7)	15.3 (0,30.5)	0.11
65 to 74	0.2 (0.2,0.2)	0.2 (0.2,0.2)	0.2 (0.2,0.2)	0.2 (0.2,0.2)	0.2 (0.2,0.2)	0.2 (0.2,0.3)	20.0 (4.7,35.3)	**0.04**
≥75	0.2 (0.2,0.2)	0.2 (0.2,0.2)	0.2 (0.2,0.2)	0.2 (0.2,0.2)	0.2 (0.2,0.2)	0.3 (0.2,0.3)	25.0 (-6.4,56.4)	0.08
Age-adjusted, by sex^a^								
Overall	0.9 (0.8,1.0)	1.1 (0.9,1.2)	1.1 (0.9,1.2)	0.9 (0.8,1.0)	1.0 (0.9,1.1)	1.1 (1,1.2.0)	18.5 (-1.3,38.3)	0.40
Men	1.0 (0.8,1.1)	1.1 (0.9,1.3)	1.1 (0.8,1.3)	0.9 (0.7,1.0)	0.9 (0.8,1.1)	1.1 (0.9,1.3)	12.2 (-11.8,36.3)	0.90
Women	0.9 (0.7,1.0)	1.0 (0.8,1.2)	1.1 (0.9,1.3)	1.0 (0.9,1.1)	1.0 (0.9,1.2)	1.1 (0.9,1.2)	24.1 (-1.0,49.3)	**0.04**

Abbreviations: ED, emergency department; CI, confidence interval.

Data from the Nationwide Emergency Department Sample and National Health Interview Survey (NHIS).

^a^ Age-adjusted to population of adults with diabetes in 2010 based on NHIS, using age groups 18–44 years, 45–64 years, 65–74 years, and ≥75 years.

Boldface indicates statistical significance (*p*<0.05)

## Discussion

Using nationally representative data, we estimated annual ED visit rates for hypoglycemia and hyperglycemic crisis among adults with diagnosed diabetes. The 2 conditions together accounted for 3.6% of all ED visits by adults with diabetes in 2011. Amid concerns about the potential hazards of overly aggressive glycemic control targets, we found that ED visit rates for hypoglycemia declined during 2006–2011. Rates for hyperglycemic crisis did not change significantly overall, although they increased for adults aged 65–74 years and for women.

Several factors may have contributed to the declining rates of ED visits for hypoglycemia between 2006 and 2011. First, glycemic control targets may have relaxed during the study period and consequently lowered the hypoglycemia incidence. Analysis of trends in national representative A1c data found that while the proportion of diabetic adults with A1c < 7% increased substantially from 1999 to mid-2000s, this proportion leveled off or decreased for some population subgroups between 2006–2010[12,13]. This change in trend could reflect adjustments to diabetes management strategies due to clinical experience or findings of studies on hypoglycemia [14–16]. Further, findings from major clinical trials [2–4] published during latter part of the study period highlighted the potential harms of hypoglycemia might have reinforced any relaxation of glycemic targets. Second, patterns of diabetes medication use may have changed, particularly as safer choices became available with pharmaceutical advances. For example, the use of sulfonylureas has declined in favor of drugs that are less likely to cause hypoglycemia, such as metformin and DPP-4 inhibitors [17,18]. Furthermore, this progress may be more attributable to oral medications than to new insulin form because, the use of the new insulin such as glargine was stable and the use of detemir doubled but remained low (16%) during our study period[19], despite that studies showed that glargine and detemir cause less hypoglycemia compared to neutral protamine Hagedorn inslulin [20–22].

The reasons why young adults aged 18–44 years had different trends in hypoglycemia from the older populations are less clear. Young adults are more likely than older adults to use insulin, but unfortunately we could not use the ED data to investigate the trends by type of medication. Also, glycemic control targets may not have relaxed for young adults compared to older adults considering their longer life-expectancy and importance of preventing later complications. This practice pattern may be related to the observed unchanging hypoglycemia rates for young adults. In fact, we found that ED visits resulting from lack of glycemic control–either hypoglycemia or hyperglycemia–were problematic for young adults. Persons aged 18–44 years have hyperglycemic crisis rate 6 to 18 times that of other age groups and high hypoglycemia rate second only to those aged 75 years or older. Ali et al also revealed that young adults were more likely to have poor A1c levels and showed no improvement in glycemic control between 1999–2002 and 2007–2010 [23]. Effective strategies to safely improve glycemic control need to be identified for this population since they will spend a greater proportion of their lives suffering from the possible consequences of poor glycemic control.

Direct comparison of our estimates on rates with other studies [24–27] is not appropriate because of differences in time periods, case definitions, and populations included. Our findings on the trends in hypoglycemia rates are similar to a prior study which showed that hospital admissions for hypoglycemia among Medicare beneficiaries declined after 2007 [28]. However, our study on ED visit rates included younger age groups and found that the trends were not improving for persons aged 18–44 years. Consistently, our study and these prior studies have all found that hypoglycemia rates were highest for people aged 75 years or older. These findings indicate, as the American Diabetes Association, the Endocrine Society and the American Geriatrics Society [9,29] recommend, it is important to set individualized glycemic goals and to provide education to patients and their caregivers on proper use of insulin, recognizing hypoglycemia, and seeking treatment to prevent hypoglycemia in older adults. On the other hand, it may be surprising that those aged 75 years or older did not have higher hyperglycemic crisis rates given the increased vulnerability their age conveys. Although the underlying reasons cannot be determined from ED data, the lower rates among those aged 75 years or older appear consistent with US trends on death rates for hyperglycemic crisis. Gregg et al found that death rates from hyperglycemic crisis in the US decreased more sharply for people 75 years or older than those aged 20–44 years over the past two decades, such that by 2010 the death rate of those 75 years or older reached 30% lower than those aged 20–44 years [30].

### Limitations

Our study has a number of limitations. First, our ED visit rates for hypoglycemia do not represent total hypoglycemia incidence because mild cases that did not require ED visit were not included. Second, because NEDS samples visits rather than patients, our results overestimated the number of people making ED visits. Third, information on type of diabetes, type of treatment, race/ethnicity, and patient fragility was not available for the ED data. Fourth, institutionalized individuals were not included in the denominators for calculating rates and thus our estimated ED visit rates may be overestimated. Fifth, because sample size in NHIS is insufficient to estimate the population with diabetes who are younger than 18 years, our study included only adults. Lastly, since our study only includes six years of data, continued monitoring is necessary to confirm trends.

## Conclusions

An important goal for diabetes treatment is to achieve euglycemia without hypoglycemia. Monitoring trends in hypoglycemia and hyperglycemic crisis provide feedback to assess glycemic control strategies. At a time when hypoglycemia from overly aggressive glycemic reduction is a serious concern, our nationally representative data suggest that national hypoglycemia rates are improving rather than worsening. Meanwhile, rates for hyperglycemic crisis remained stable. Given the continued development of treatments that bring both benefits and risks, it is important to continue to monitor these trends over time. Our findings also suggest that future efforts to prevent these complications should target on the susceptible population of adults aged 18 to 44 years and those aged 75 years or older.
